# Supplementary material for: The organizational production of earnings inequalities, Germany 1995–2010
Source: PLoS One. 2020 Sep 9;15(9):e0237970. doi: 10.1371/journal.pone.0237970 (PMC7480845; doi:10.1371/journal.pone.0237970)
Supplement: S1 File — (DOCX) [file pone.0237970.s001.docx]

**S1 Imputation strategy for top coded income**

In the German social security system records earnings are censored at the social contribution limit which differs by year and in East and West Germany. The wage information stated in the social security notification is yearly sum of wages related to the employment episode. The daily wage is the episode wage divided by number of calendar days, i.e. it is the average wage for this period. Wages are deflated by the CPI, base year is 2010. We consider all daily wages as censored that are larger than the contribution limit minus 3 € in order to account for rounding errors.

**Imputation methods**

1.1 Simple. A simple approach to top-coding is to use individual information only to impute missing earnings for top coded cases. This is the most common approach in the literature that developed out of top coded survey data. Since much wage variation is associated with employment organization this is not a satisfying approach for linked employer-employee panel (LEEP) data.

1.2 Card/Heining/Kline

To improve top-code estimates Card, Heining and Kline (4) include in their imputation equations using German LEEP data the leave-one-out mean workplace and mean individual wage and workplace shares of censored observations in their imputation equations. In our replication of their imputation model we discovered that their method produces too few jobs immediately to the right of the censoring limit and too few cases in the far tails of the distribution.

1.3 The lagged wage alternative

Rather than focusing on the mean individual and workplace wage prior to the observation year we utilize information on lagged wages in our prediction equation. When the lagged wage is missing it is imputed in an earlier stage. We reason that the censored wage is more strongly influenced by the most recent wage than by mean wages over longer periods.

1.3.1 Individual Stratification

Individual variables are used in the imputation models to stratify the sample. These include the cross-classification of gender, education, age groups and East and West German residence. Each imputation equation is estimated separately for each cell of this cross-classification.

1.3.2 Firm variables

Each imputation is based on a prediction equation that contains lagged individual daily earnings plus a series of organizational characteristics.

We include the following workplace level variables in the imputation equations: fraction of workers with university degree, mean years of schooling of firm by gender, log firm size, fulltime employees, log firm size squared, dummy for firm size>10 full-time employees, mean log real daily wage of co-workers, fraction of co-workers with censored wage, dummy firm has only 1 worker in current year.

1.4 Limitations

This imputation is designed to produce a single point estimate or each top coded case. While this is unlikely to be a problem in very large samples, such as ours or Card et al (2013), future applications should consider recognizing uncertainty in the imputation with some form of multiple imputation strategy.

1.5 Stata Code for Imputation Procedure

Since censored wages are likely to be censored in prior years we utilize an iterative imputation strategy.

### Step 1: independent imputation models by year, age (4 categories) education (five categories), sex and east/west Germany

$${logw}_{it}=\gamma*x_{it}+\varepsilon_{it} t\in\left[ 1994\ldots2010 \right]$$

Predict ${logw}_{it-1}^{ind}$

Step 2: include lagged variables in order to account for correlation over time

$${logw}_{it}=\propto*{logw}_{it-1}^{type}+ \beta*\left( {logw}_{it-2}^{type} {-logw}_{it-1}^{type} \right)+\gamma x_{it}+\varepsilon_{it}$$

$$t\in\left[ 1996\ldots2010 \right]$$

type = {org, ind, lag, mean}

| If person in sample in t-1 | org = observed uncensored wage  ind = imputed wage from regression in t-1 without lag variables  lag = imputed wage from regression in t-1 with lag variables |
| --- | --- |
| If person is not in sample in t-1 | ${logw}_{it-1}$ is replaced by the person mean estimated:  ${logw}_{it-1}^{mean}={logw}_{it}^{org, ind}-mean({logw}_{it}^{org, ind}-{logw}_{it-1}^{org, ind})$  i.e. current wage – average change  $\left( {logw}_{it-2}^{type} {-logw}_{it-1}^{type} \right)=mean({logw}_{it-2}^{org, ind}-{logw}_{it-1}^{org, ind})$  i.e. average change 🡪close to 0 |

| If person is not in sample in t-2 | ${logw}_{it-1}$ is replaced by mean  ${logw}_{it-1}^{mean}={logw}_{it}^{org, ind}-mean({logw}_{it}^{org, ind}-{logw}_{it-1}^{org, ind})$  i.e. current wage – average change  $\left( {logw}_{it-2}^{type} {-logw}_{it-1}^{type} \right)=mean({logw}_{it-2}^{org, ind}-{logw}_{it-1}^{org, ind})$  i.e. average change 🡪close to 0 |
| --- | --- |
