# Supplementary material for: The organizational production of earnings inequalities, Germany 1995–2010
Source: PLoS One. 2020 Sep 9;15(9):e0237970. doi: 10.1371/journal.pone.0237970 (PMC7480845; doi:10.1371/journal.pone.0237970)
Supplement: Attachment — Submitted filename: Response to Reviewers.docx [file pone.0237970.s002.docx]

Dear Reviewers

Thank you for a very insightful pair of reviews. We have changed the paper in multiple ways as a result. We outline these in point-by-point responses to the reviewers. Not in response to the reviewers, but to simplify the exposition, we dropped the analysis of segregation effects on internal inequalities. As you may recall from the last version these effects were quite weak, and added a great deal of complexity to an already too complex analysis.

Reviewer 1

1. The reviewer points out that the FE models really focus on within workplace change and suggest we move into a hybrid HLM framework. This turned out to be a very productive suggestion, as doing so revealed that 90% of the workplace variance in mean wages was a stable between workplace characteristic. It was 75% for within workplace inequality. This led us to both a somewhat different interpretation of the overall statistical exercise and to add a direct modeling of the impact of the birth and death of workplaces on the between and within components. We found that new firms pay mean wages 9% lower than more long lived firms, and 4% lower than firms that exit the panel. The change to a hybrid model also led to a series of qualifications in the hypotheses as to whether they applied primarily to between or within workplace inequalities, which was a very useful exercise.
2. We now include more detail on how the industry fixed effect analysis tied to hypothesis 1 is estimated, and have dropped the figure in favor of tabular presentation. We have added a parallel analysis of within workplace inequality here as well.
3. The reviewer also wondered if rather than reflecting the causal force of industry that the observed between industry wage polarization might reflect something correlated with industry. We agree and now make clearer that we see two processes underlying this result: increased market power associated with high wage firms and the outsourcing of lower paid jobs to new low wage firms in different industries. Neither market power or outsourcing are observed, but rather are the mechanisms pointed to in the literature. We make this distinction between observed effects and unobserved mechanisms clearer throughout the manuscript. We appreciate the reviewer’s suggestion to design a comparative industry study and will implement it in a future paper.
4. Reviewer 1 points out that the literature review adequately motivates study and so we do not need Figure 1. We have dropped that figure.
5. We now make clear that we use the variance in logged earnings because it has become standard in this literature, but in the conclusion suggest that future research might use other measures sensitive to particular positions in the income distribution like 90/10 ratios or quantile regression.

Reviewer 2

1. Points out that we do not distinguish between human capital and relational inequality theories, or connect the empirical results back to these theories. We think the last version of the paper was confusing in this regard. Our core use of HC and RIT is to build a reasonable set of control variables as a robustness test of the more basic proposition that changes in firm job structure are producing rising inequalities. We have reorganized the paper to reflect this distinction more clearly. It is also the case that we do not see HC and RIT as competing, but rather complementary, descriptions of plausible processes in workplace level wage setting. We do comment on the importance of both educational levels and gender composition for explaining both between and within workplace inequalities in the discussion section, but these are not our primary focus.
2. The conclusion now includes a more critical discussion of the limitations of our analysis and the types of research that would be useful going forward.
3. Reviewer 2 points out that we do not have a direct measure of new non-union firms and that this is a prominent alternative or complementary explanation of rising between workplace inequality trends. While we do not know the union status of workplaces, we have now included an indicator of new firms (those that enter our panel after 1995). This does not change other results, but is a result in its own right – new firms pay lower wages than either continuing firms or those that exist the panel.
4. Reviewer 2 wonders if the under sampling of large firms for confidentiality reasons leads us to under observe potential outsourcing firms. All of our analyses use sampling weights to adjust the final sampling estimates to correct for this initial under sampling, so the answer is probably not.
5. Reviewer 2 wants to know more about the self-employed in German, who are excluded from our analyses. We have added descriptive material on the size and earnings of the self-employed in Germany in our discussion of sample exclusions.
6. The implications of part-time as a potential source of power differentials between and within firms is now much clearer in the paper. This reflects are move to hybrid models, which reveals that more part-time labor undermines full-time wages between firms, while supporting wage gains for full-time labor within firms. This result is novel and an important adjudication between the dualization and worker power predictions. We agree that the growth in part-time jobs is also associated with industry and firm segmentation, and this is one of the reasons why we control for industry in the final model and hypothesize the dualization effect within workplaces.
7. Reviewer 2 points out that union membership is not time stable. We now explain that we see it as stably associated with firms. We see the addition of a new set of indicators for establishment births and deaths as partial controls for omitted variable bias. We have also redesigned the analysis to add industry as a control variable, which should perform a similar function.
8. Smaller comments
   a. We now point our earlier in the manuscript that we do not have an indicator of fixed-term contracts.
   b. We use variance in logged earnings as our main inequality measure because it is standard in this literature. We do not pursue an analysis of alternative measures, such as the gini. We do point out in the conclusion that such analyses might be informative, although we stress distributional analyses such as the 90/10 ratio or quantile regression approaches.
