# Supplementary material for: The organizational production of earnings inequalities, Germany 1995–2010
Source: PLoS One. 2020 Sep 9;15(9):e0237970. doi: 10.1371/journal.pone.0237970 (PMC7480845; doi:10.1371/journal.pone.0237970)
Supplement: Attachment — Submitted filename: Response to Reviewers.docx [file pone.0237970.s003.docx]

Response to Reviewers

Reviewer 1 suggests a more clear statement of hypotheses and results in the abstract and one improvement in text.

These were great suggestions and we have changed the abstract as suggested and on page four changed “establishment level internal simplifications” to “reductions in occupational skill complexity”

Reviewer 2 asks “ Do you adjust for the fact that these values are imputed, for example by adding uncertainty by means of random draws from the corresponding error distribution? If not, I would at least critically mention this in the main text.”

We do not adjust for this uncertainty in our estimates, nor did previous research. It is an important technical point, and we will consider this as an improvement to future iterations of our imputation strategy. We now note in Supplemental Information text that this is a limitation:

1.4 Limitations

“This imputation is designed to produce a single point estimate or each top coded case. While this is unlikely to be a problem in very large samples, such as ours or Card et al (2013), future applications should consider recognizing uncertainty in the imputation with some form of multiple imputation strategy.”

Reviewer 2 suggests dropping Table 3 and 4 footnotes referring to Rho statistics. These footnotes refer to a null model not reported in the table not the reported estimates and so we prefer to keep them.

Finally, reviewer 2 asks: You include year fixed effects in all the models but do not interpret them. What do they tell us?

For Table 3 we add this interpretation: “We also note from the baseline model that mean workplace inflation adjusted earnings in Germany barely changed over time, rising slowly through 2001 and declining thereafter.”

For Table 4 : “The time trend reported in the baseline model confirms that average within workplace inequality has been rising across the study period.”
